# Diagnostics of Mutations in MMR/*EPCAM* Genes and Their Role in the Treatment and Care of Patients with Lynch Syndrome

**DOI:** 10.3390/diagnostics10100786

**Published:** 2020-10-05

**Authors:** Joanna Sobocińska, Tomasz Kolenda, Anna Teresiak, Natalia Badziąg-Leśniak, Magda Kopczyńska, Kacper Guglas, Anna Przybyła, Violetta Filas, Elżbieta Bogajewska-Ryłko, Katarzyna Lamperska, Andrzej Mackiewicz

**Affiliations:** 1 Department of Cancer Immunology, Chair of Medical Biotechnology, Poznan University of Medical Sciences, 8 Rokietnicka Street, 60-806 Poznan, Poland; kolenda.tomek@gmail.com (T.K.); mg.kopczynska@gmail.com (M.K.); przybyla.anna.ump@gmail.com (A.P.); mackiewicz.aa@gmail.com (A.M.); 2 Department of Diagnostics and Cancer Immunology, Greater Poland Cancer Centre, 15 Garbary Street, 61-866 Poznan, Poland; 3 Laboratory of Cancer Genetics, Greater Poland Cancer Centre, 15 Garbary Street, 61-866 Poznan, Poland; anna.teresiak@wco.pl (A.T.); kacper.guglas@gmail.com (K.G.); kasialam@o2.pl (K.L.); 4 Oncological Genetics Clinic, Greater Poland Cancer Centre, 15 Garbary Street, 61-866 Poznan, Poland; badziag.natalia@gmail.com; 5 Postgraduate School of Molecular Medicine, Medical University of Warsaw, 02-091 Warsaw, Poland; 6 Department of Tumor Pathology and Prophylaxis, Poznan University of Medical Sciences, Greater Poland Cancer Centre, 15 Garbary Street, 61-866 Poznan, Poland; vfilas@ump.edu.pl (V.F.); elzbieta.bogajewska-rylko@ump.edu.pl (E.B.-R.); 7 Department of Cancer Pathology, Greater Poland Cancer Centre, 15 Garbary Street, 61-866 Poznan, Poland

**Keywords:** Lynch syndrome, hereditary cancer, colorectal cancer, MMR, diagnostics, IHC, MSI, NGS, MLPA

## Abstract

Lynch syndrome (LS), also known as hereditary nonpolyposis colorectal cancer (HNPCC), is a disorder caused by an autosomal dominant heterozygous germline mutation in one of the DNA mismatch repair (MMR) genes. Individuals with LS are at an increased risk of developing colorectal and extracolonic cancers, such as endometrial, small bowel, or ovarian. In this review, the mutations involved with LS and their diagnostic methods are described and compared, as are their current uses in clinical decision making. Nowadays, LS diagnosis is based on a review of family medical history, and when necessary, microsatellite instability (MSI) or/and immunohistochemistry (IHC) analyses should be performed. In the case of a lack of MMR protein expression (dMMR) or MSI-H (MSI-High) detection in tumor tissue, molecular genetic testing can be undertaken. More and more genetic testing for LS is based mainly on next-generation sequencing (NGS) and multiplex ligation-dependent probe amplification (MLPA), which provide better and quicker information about the molecular profile of patients as well as individuals at risk. Testing based on these two methods should be the standard and commonly used. The identification of individuals with mutations provides opportunities for the detection of cancer at an early stage as well as the introduction of proper, more effective treatment, which will result in increased patient survival and reduced costs of medical care.

## 1. Introduction

Colorectal cancer (CRC) is currently one of the most commonly diagnosed cancers, taking third place in men and fourth in women. Only in 2018, more than 1.8 million new cases and almost 900 thousand deaths were recorded worldwide. Approximately 70–80% of them are sporadic cancers, while genetic factors are responsible for the remaining 20–30% of known cases [1,2]. In people with genetic load, familial cancer syndromes, such as Lynch syndrome (LS) or familial adenomatous polyposis, are most commonly the culprits of cancer development. LS, also known as hereditary nonpolyposis colorectal cancer (HNPCC), is associated with 3–4% of hereditary cancers, while familial adenomatous polyposis-approximately 1% [3].

LS is characterized by a predisposition to a spectrum of cancers, mainly colorectal and endometrial cancer. It is associated with autosomal heterozygous germline mutations in either one of the DNA mismatch repair system (MMR) genes-*MLH1*, *MSH2*, *MSH6*, *PMS2* or with the epithelial cell adhesion molecule (*EPCAM*) gene [4,5]. Mutations in *MLH1* and *MSH2* are the most common ones and represent approximately 80–90% of all cases, while the other 10–20% applies to mutations in *MSH6* and *PMS2* genes. Mutations in *EPCAM* are rare and constitute about 3% of cases [5].

In cells, in which a malfunction of the MMR system is observed, mutations usually occur in short tandem repeats (STRs), and the said mutations are referred to as microsatellite instability (MSI) [6,7]. Carriers of germline mutations in MMR genes display an 80% risk of developing cancer by the age of 70, and the average age of onset in LS is 45, compared with an average of 60 in sporadic CRC. LS is usually divided into two types—I and II [8].

### 1.1. LS I-Clinical Presentation

Colorectal cancer is the main type of cancer observed in LS patients. Individuals with LS are also at increased risk of developing synchronous (primary tumors diagnosed within six months of each other) and metachronous (primary tumors occurring >6 months apart) cancers. The risk of CRC development is significantly increased in patients with mutations in *MSH2* and *MLH1* genes compared with ones with *MSH6* and *PMS2* mutations. CRCs in LS are predominantly right-sided mucinous tumors [9,10,11], usually occurring at a young age [10] and evolving from pre-existing adenomas, which more likely and more rapidly progress to cancer in people with LS than in people with sporadic adenomas (2–3 vs. 8–10 years) [7]. Histologically, these cancers are poorly differentiated, which makes the identification difficult [10,12].

### 1.2. LS II-Clinical Presentation

Individuals with LS are at an increased risk of developing not only CRC but also extracolonic cancers, like endometrial, ovarian, stomach, small intestine, urinary tract, pancreatic, brain, and that of the cutaneous sebaceous glands also known as Muir–Torre syndrome. Endometrial cancer (EC) is the most common extracolonic cancer and occurs with similar frequency to CRC in women [11,12]. However, the risk of developing EC in patients with LS by the age of 70 ranges from 14% to 71% and is dependent on a mutation in a particular gene-approximately 14–54% for patients with *MLH1/MSH2* mutations, 17–71% for patients with a mutation in *MSH6* and 15% in for instances of *PMS2* [13]. Interestingly, in women with an *MSH6* mutation, EC will more likely develop than CRC [14]. Moreover, EC associated with Lynch syndrome is usually located in the low uterine segment and it is mostly observed in individuals with an *MSH2* mutation [15,16,17]. For comparison, the risk of developing ovarian cancer is much lower (4–20%) [13], and is also associated mostly with a mutation in the *MSH2* gene [14].

**Figure 1:**
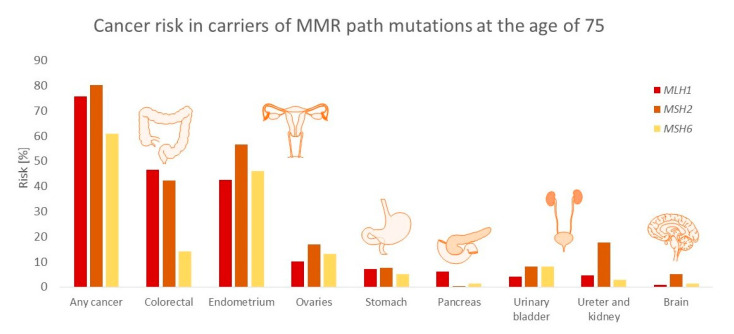
Cancer risk in carriers depending on the mutated mismatch repair (MMR) path gene (*MLH1/MSH/MSH6*) at the age of 75. The risk of endometrial and ovarian cancers was calculated in females, all others-in both sexes [16].

The risk of developing small bowel cancer ranges from 0.4% to 12%, and the average age of onset is 46–49 vs. 50–70 in the overall population [13,18]. The tumor is usually located in the duodenum and jejunum, less frequently in the ileum, and is mostly observed in individuals with a mutation in *MLH1* [19]. The risk, depending on the cancer type and MMR mutation, is presented in Figure 1.

## 2. Function and Mutations in Genes Responsible for DNA Repair and Involved in LS

**Figure 2:**
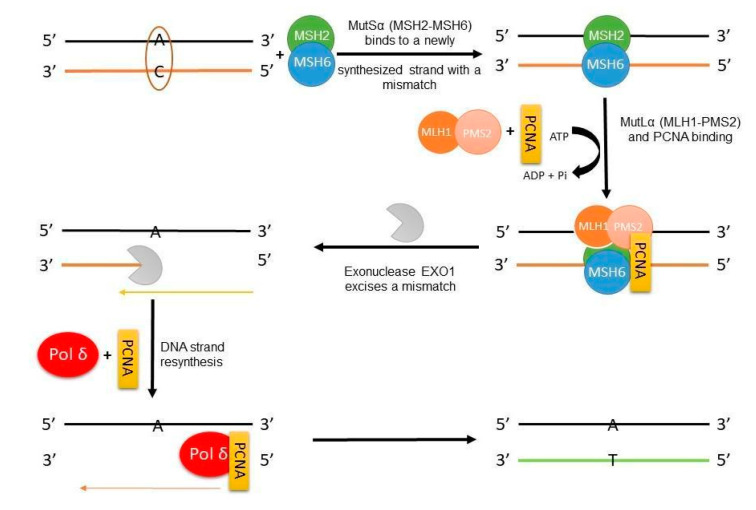
The MMR mechanism in a eukaryotic cell on the example of a single-base mismatch. PCNA—proliferating cell nuclear antigen; Pol δ—polymerase delta.

The DNA mismatch repair system enhances genome stability by recognizing and repairing polymerase errors. In LS, germline variants are mutated in the genes that encode MMR proteins. Mutations in MMR proteins result in microsatellite instability, which is observed in some cancers, including those associated with LS. Originally, the MMR system was identified and characterized in *Escherichia coli*, and it requires MutS, MutL, and MutH proteins. In humans, the mismatch repair system consists of six MMR proteins, MLH1, PMS2, MSH2, MSH6, MSH3, and PMS1, which are *E. coli* protein homologs [20,21]. The mechanism of their role in DNA repair is presented in Figure 2.

The mechanism of mismatch base repair in eukaryotic cells is initiated by a MutSα (MSH2-MSH6) heterodimer, which recognizes mismatched single bases or small insertions/deletions (ID) (1–2 nucleotides long), or by the MutSβ (MSH2-MSH3) heterodimer, which recognizes larger insertions/deletions, up to 16 nucleotides. Both of the mentioned heterodimers are ATPases, and this activity is crucial for proper initiation and recognition [20,21]. Through ATP-dependent activation, MutS undergoes a conformational change into the sliding clamp, which moves along the DNA [22], and this state allows further recruitment and activation of the MutLα complex (MLH1-PMS2). PMS2, included in MutLα, is an endonuclease dependent on the PCNA (proliferating cell nuclear antigen) and RFC (replication factor C) (PCNA activates MutLα endonuclease activity). The complex MutLα is responsible for the mismatched base’s incision and, thus, has an essential role in MMR. Interestingly, the endonuclease activity of MutLα is essentially required in 3′ nick directed excision and is not obligatorily needed in 5′ nick mismatch excision [20,21,22,23,24].

In 3′ nick directed excision, the MLH1-PMS2, with the help of PCNA, initiates the incision of the strand in 3′ heteroduplex, resulting in a strand break usually 5′ from the mismatch, which is later used as a starting point for the EXO1 (exonuclease 1) 5′→3′ excision of the intermittent strand with the included mismatch, so in the next step, polymerase δ, after binding with PCNA, can re-synthesize the correct DNA strand, Figure 2 [20,21,22,23,24]. When a gap is located 5′ to the mismatch, the interaction between MutS, EXO1, and replication protein A (RPA) is needed. The MutS activates the EXO1, and RPA stimulates the whole process. Nevertheless, MutLα might play a role in this excision as well, although a non-compulsory one. It is suspected that the MLH1-PMS2 complex takes part in the termination of the 5′ excision right after cutting the mismatch [20,21,22,23,24,25].

However, besides the EXO1-dependent MMR, there is also the possibility of an EXO1-independent pathway, where repeated strand breaks by MutLα might be required. That would further lead to the production of 3′ ends or the excision of a newly synthesized strand near the mismatch, then directly to polymerase δ binding and later to displacement strand resynthesis [24,26]. Aside from the role played by MutLα in DNA MMR, it also participates in cell damage signaling, the control of the cell cycle checkpoint, and directing the cell into the apoptosis pathway [20,21]

Mutations in MMR genes, *MLH1*, *PMS2*, *MSH2*, *MSH6*, are associated with LS [27,28]. A loss of MMR system functions results in the accumulation of mismatches in microsatellite sequences, which is referred to as MSI [29]. MSI is observed in over 90% of colon tumors in LS patients and only 10–15% in patients with sporadic CRC [13]. In the second case, the presence of microsatellite instability is due to the hypermethylation of the *MLH1* promoter and not due to a mutation in the germline [13,30].

Mutations of *MSH2* and *MLH1* are the most frequent due to their obligatory functions in the MMR system. The most commonly observed types of mutations in these genes are nonsense mutations, in which the substitution of a single base leads to the appearance of a stop codon and results in the reduction of a polypeptide chain (the protein is usually shortened and nonfunctional). Additionally, missense mutations can also be observed [31]. A significant part of the mutations in the MMR genes is unique, characteristic for the family. However, many mutations are already known and are quite commonly observed in LS patients [32].

According to the InSiGHT database, there are over 3000 different variants of MMR genes that predispose LS: *MLH1* mutations constitute 40% of the variants, *MSH2*-30%, *MSH6*-20% and *PMS2*-10%. The type of mutations mostly observed in these genes are point mutations, and quite often-significant rearrangement, deletions or insertions, are as well. According to Knudson’s two-hit hypothesis, both copies of the MMR gene have to be inactivated for the tumor’s phenotype manifestation [33]. In an LS-associated cancer, the first hit mutation is usually an inherited point mutation or a massive rearrangement, and the second-a loss of the wild-type allele or gene conversion. Recently, it was observed that constitutional epimutations could serve as the first hit mutation and promoter methylations as the second [34]. 

In LS, the most common constitutional epimutation is the hypermethylation of the *MLH1* promoter in one of two alleles, which leads to silenced gene expression in most somatic tissues and the hypermethylation of *MSH2* caused by *EPCAM* gene mutation [34]. The LS families, in which no mutation in MMR genes’ sequence has ever been previously observed, need to be diagnosed for epigenetic mutations [34].

The epithelial cell adhesion molecule (EPCAM/CD326) is a 39–42 kDa transmembrane glycoprotein almost exclusively expressed in epithelial tissue and epithelial-derived cancers and functions not only in cellular adhesion but also signaling, migration, proliferation and differentiation [35]. Due to a high and stable expression of EPCAM in primary cancers, adenocarcinomas, metastases and malignant effusions and cancer stem cells, including circulating cancer stem cells, it could be used as a biomarker [35]. The expression of EPCAM can be regulated by epigenetic mechanisms, for example, DNA promoter hypomethylation, as well as elements of signaling pathways, for example, the WNT signaling pathway [36]. Proteolytic changes in the transmembrane EPCAM protein leads to the release of extra- and intracellular domains. The cytoplasmic form creates a transcriptional complex with WNT signaling and influences genes connected with cell proliferation and stemness maintenance. The second component, extracellular form, functions as a ligand, which stimulates, for instance, PI3K/AKT/mTOR pathways and supports cancer cell growth [36].

Mutation in *EPCAM* genes can cause two different, unrelated diseases such as LS or congenital tufting enteropathy (CTE), which depends on the type and nature of changes [37]. The *EPCAM* gene is located 15 kb upstream from the *MSH2* gene and deletions of the 3′ end of the *EPCAM* gene, including its polyadenylation signal, first cause changes and eventually the inactivation of the promoter of *MSH2*, while maintaining the expression of *EPCAM* [38]. It should be noted that these changes are not to be observed in the promoter or start site of *MSH2*, but only in the polyadenylation signal of the *EPCAM* gene [39]. It was found that twenty-five 3′ deletions of *EPCAM* at the sequence level are implicated in causing LS. Most of these deletions are in exons 8 and 9 and are mediated by a recombination between imperfectly homologous Alu repeats [39]. These mutations could cause the loss of the intracellular element of EPCAM without changes in the transmembrane, as well as extracellular domains. However, it is unclear if these truncated EPCAM proteins are created because *EPCAM-MSH2* fusions are indicated [37].

Rumilla et al. checked the frequency of deletions of *EPCAM* (TACSTD1) in MSH2-associated LS cases. They indicated that 20% to 25% of cases possess deletions that were suspected of having a mutation in *MSH2*, but in which a germline mutation was not detected [40]. In the case of CRC connected with *EPCAM* deletions, the observed risk of disease is comparable to that of *MSH2* mutation. Contrary to this, the risk for endometrial cancer is lower compared to *MSH2* mutation carriers. It should, however, be noted that this depends on the size and location of the *EPCAM* deletion [38]. In CTE, 19 different *EPCAM* mutations were indicated and they were grouped to chromosomal deletions, non-coding/splicing, frameshift/truncation and missense type. The frameshift mutation c.499dupC is the most important from a clinical point of view [37]. The detailed characteristics of *MSH2, MLH1, MSH6,* and *EPCAM* is presented in Table 1.

## 3. Diagnostics of LS

The diagnostics, treatment, and care of patients with LS should differ from methods used in patients with sporadic colorectal cancer, due to genetic and clinical differences of these disease types. Efficient and cost-effective diagnostics of people with suspected LS is crucial to implement an appropriate prevention program or treatment regimen [50].

Diagnostics begin with the identification of families at high risk for cancer through family history and other distinguishing features, such as early age of onset or metachronous disease. The *Amsterdam criteria* and the *Revised Bethesda Guidelines* are used in traditional testing for initial identification, Table 2. When a patient fulfills all three of the *Amsterdam criteria* or at least one criterion of the *Revised Bethesda Guidelines*, then microsatellite instability testing or/and immunohistochemistry (IHC) testing should be performed [13,51]. However, a universal strategy is becoming more commonly used, and it involves screening all individuals with newly diagnosed CRC with a tumor testing (MSI/IHC) [13,52]. In a patient with an MSI-H (MSI-High) tumor and/or lack of expression of one of MMR proteins, further genetic testing for the identification of a specific mutation or epimutation causing LS should be performed. Such families should receive comprehensive care focused on early diagnosis, which would enable earlier treatment and, at the same time, increase the survival rate among high-risk patients [13,51].

### 3.1. Clinical Diagnostics

Clinical diagnostics of LS are mainly based on the *Amsterdam criteria I* and *II*, and the *Revised Bethesda Guidelines*, Table 2. The application of the *Amsterdam criteria I* involves recognition of patients at a higher risk of developing LS-associated colorectal cancer based on patients’ family history and clinical evaluation [7,13]. However, the *Amsterdam criteria I* was revised due to the subsequent discoveries of extracolonic cancers also being associated with LS. From now on, the *Amsterdam criteria II* also included the occurrence of LS-associated extracolonic cancer [13,53].

Nevertheless, several studies reveal the low sensitivity (22%) and specificity (98%) of the *Amsterdam criteria*. For this reason, new criteria for the identification of LS patients were introduced, namely the *Revised Bethesda Guidelines*. In patients meeting at least one of the guidelines, screening for mutation carriers in *MLH1, MSH2, MSH6,* or *PMS2* genes is performed by way of immunohistochemistry and/or microsatellite instability testing [13]. It is also recommended to perform both screening tests also in patients’ first degree relatives [55]. Unfortunately, both the *Amsterdam criteria* and the *Revised Bethesda Guidelines* are not sensitive enough to detect all patients with LS. The patients’ pedigree is not always reliable or available. Furthermore, not every patient with LS fulfills all of the *Amsterdam criteria* [7,56]. Adar et al. noted that the *Amsterdam II criteria* and the *Revised Bethesda Guidelines* would have missed nearly 62.5% and 50% of the LS cases in the study, respectively, and also recommend future development of a universal screening program for CRCs as well as ECs, which would increase the identification of LS [57].

Additionally, clinical prediction models are also in use, such as *MMRpredict*, *MMRpro*, *Prediction of Mismatch Repair Gene Mutations in MLH1*, *MSH2*, and *MSH6* (*PREMM 1, 2, 6*) [13,58] or *PREdiction Model for gene Mutations (PREMM5)*, which may be considered as PREMM1,2,6 replacement [59]. Their function is to determine the risk of carrying mutations in MMR genes in individuals with suspected LS. The aforementioned prediction models are useful when the suspected individual is unaffected, or the performance of MSI testing is impossible [60]. Thus, they can also be used as one of the criteria allowing further MSI/IHC testing when the calculated risk is ≥5% [13,60] or ≥2.5% in PRIMM5 [59]. The used clinical criteria, function, sensitivity, and specificity of the models are presented in Table 3.

### 3.2. Immunohistochemistry and Microsatellite Instability Testing

Before performing costly molecular testing in individuals with suspected LS, it is recommended to undertake screening tests first, which may suggest the probability of MMR gene mutation. MSI and IHC are used to identify patients at high risk of LS for further genetic testing [7]. The screening tests are based on tumor testing performed on formalin-fixed tissue from surgical specimens [61].

Immunohistochemistry can directly indicate a lack of a particular MMR protein expression, saving time and costs of testing other MMR genes. IHC uses primary monoclonal antibodies directed against specific proteins, in diagnostics for LS, against MSH2, MLH1, MSH6, and PMS2 proteins. The sensitivity and specificity of immunohistochemistry are approximately 83% and 89%, respectively [7,13,61,62].

**Figure 3:**
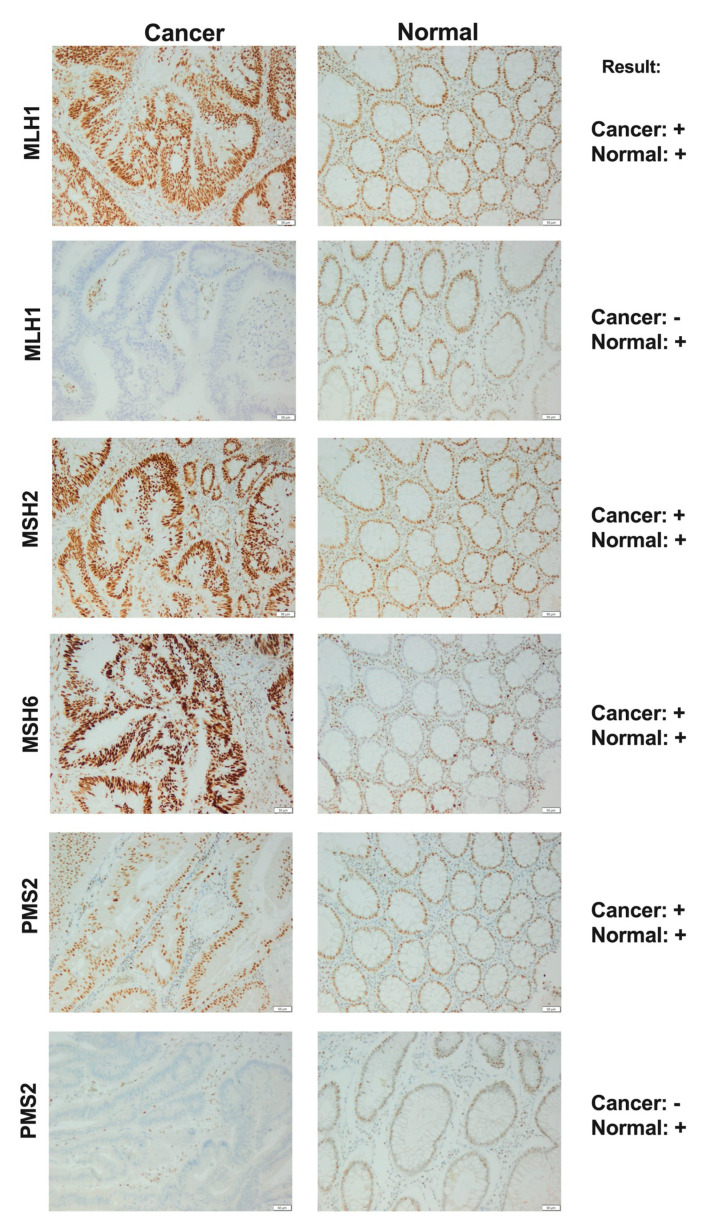
Immunohistochemistry staining of MLH1, MSH2, MSH6 and PMS2 proteins in colon cancer and normal tissue as the example of a diagnostic panel. The scale bar represents 50 μm; “+”—positive staining; “−”—negative staining.

MSH2 and MLH1 are obligatory proteins in the MMR system and they dimerize with secondary proteins-MSH6 and PMS2, respectively. When the mandatory protein is defective, the whole heterodimer becomes unstable, and the secondary protein becomes degraded. On the other hand, when the secondary protein is altered, the heterodimer can remain stable since obligatory proteins can also form heterodimers with other secondary proteins (MSH3, MLH3, PMS1) [7,61]. Shia et al. [61] showed that in IHC testing, a 2-antibody panel (MSH6 and PMS2) might be as predictive as a widely used 4-antibody panel (MSH2, MLH1, MSH6, PMS2), Figure 3. The antibody against MSH6 can detect a loss of protein expression both in MSH6 and MSH2, and the antibody against PMS2-in both PMS2 and MLH. The proposed approach may increase cost-effectiveness in an already widely used screening method. However, the 2-antibody panel should be used only as a first-line screening method and if any abnormality is detected, the secondary IHC with the use of additional antibodies should be undertaken. 

When it comes to the interpretation of IHC results, a loss of expression of the MMR protein is confirmed based on the complete absence of staining in the tumor tissue. However, in some cases, a heterogeneity of staining can be observed. This includes both areas with weak and thus uncertain staining as well as areas with both strong and no staining. Such heterogeneity can create false positive or false negative results [63,64,65]. The possible IHC results and their interpretations are presented in Table 4.

Microsatellite instability testing is also a screening test performed on patients who fulfill the *Amsterdam criteria I or II* or the *Revised Bethesda Guidelines*, or due to the universal screening strategy-all new patients affected by CRC [13,52,66].

MSI can be used for the identification of tumors caused by a defective MMR system. Microsatellites are the DNA stretches especially susceptible to acquiring errors when the MMR system is faulty. The identification process is based on analyzing the difference in the length of microsatellite repeats in tumor tissue compared to the healthy non-neoplastic tissue surrounding the tumor. Generally, panels of 10 markers [67] and commercially available kits [68,69] are in use, as well as a panel proposed by Bethesda [7,70,71]. 

Before DNA amplification, the microdissection of examined tissue must be performed. Microsatellite instability is considered to be present when, in DNA samples from tumor tissue, an additional amplicon of a different size is observed, compared to DNA samples from healthy tissue from the same patient. The additional amplicon indicates insertion/deletion in the examined microsatellite sequence and its length is changed. When ≥30% of the markers are unstable, the tumor is considered MSI-H, when less than 30% of the markers are unstable, the tumor is considered MSI-L (MSI-Low). When no additional amplicons are observed, the tumor is considered microsatellite stable (MSS). Nevertheless, it was also noted that microsatellite instability could be stated if at least one mononucleotide marker is unstable, which can reduce the number of markers in the panel and makes all diagnostic processes more cost-effective [7,62,72].

Alternatively, microsatellite testing can also be carried out using next-generation sequencing (NGS). No healthy tissue is needed as a benchmark in this method, which makes it possible to perform MSI testing even in individuals from whom obtaining healthy tissue is problematic or impossible. MSI-NGS can easily be included in panels already used in molecular diagnostics, which would reduce both the number of tests and overall costs [73].

Sometimes, in tumors in patients with LS-associated cancer, all four MMR proteins are present, but nevertheless microsatellite instabilities are observed. This phenomenon can be explained by the type of mutation in the altered gene. In this case, the expression of a stable protein with a regular epitope, although unfunctional, is observed [7,72].

When MSI-H and lack of expression of both *MLH1* and *PMS2* are observed, tumor *BRAF* V600E mutation and/or *MLH1* promoter hypermethylation testing is recommended to distinguish sporadic colorectal cancer from CRC caused by an MMR defective system [13,52,62,66].

*BRAF* mutation analysis is performed using immunohistochemistry and the VE1 antibody, the PCR reaction, or exon 15 sequencing. *BRAF* testing is widely used in LS diagnostics since the V600E mutation does not occur in LS patients but it is observed in half of the patients with sporadic CRC [74,75]. When *BRAF* mutation occurs, no further diagnostics for LS is necessary (LS excluded). If there is no *BRAF* mutation in a tumor, performing further genetic tests for *MLH1* and *PMS2* germline mutations is recommended [76]. The sensitivity and specificity of *BRAF* V600E IHC are 69 and 99%, respectively [77].

Besides *BRAF* mutation analysis, *MLH1* promoter methylation also can be performed if MLH1 and PMS2 proteins are absent. If the hypermethylation and no *BRAF* mutation occur in the tumor tissue, it is recommended to analyze *MLH1* promoter methylation results in the healthy tissue to determine epimutation. Epigenetic mutations that cause LS are rare but may be present in both tumor and healthy tissue [13].

Microsatellite instability in tumor tissue is evidence of MMR germline mutation. However, through MSI testing, a specific altered gene cannot be determined. It is also impossible to distinguish between LS-associated and sporadic cancer. On the other hand, immunohistochemistry allows to identify the specific, mutated gene in the examined individual but does not differentiate a somatic and germline mutation. Therefore, using both methods guarantees extensive and reliable screening diagnostics for patients with LS [55,78]. However, if only one of the tumor tests can be performed, the choice between MSI and IHC should depend on the preferences of the physician, staff opinion, or the available technologies [79]. The MSI and IHC diagnostics are significant also because of the different responses to treatment of dMMR (MMR-deficient)/MSI tumors. In patients with dMMR/MSI tumors, distant and local lymph node metastases are rarer and fewer advanced stage tumors are observed [5,72].

### 3.3. Molecular Testing

Further molecular diagnostics should be performed in patients and their families in which MSI-H and/or lack of expression of at least one MMR protein were observed. The molecular diagnostics should be complex because none of the methods can detect all types of possible mutations in MMR/*EPCAM* genes. When the family mutation is known, the appropriate diagnostic method should be used, depending on the type of mutation. If a mutation is unknown, methods that enable gene scanning should be used. Gene scanning methods for unknown mutations and their mechanisms are presented in Table 5. Currently, next-generation sequencing (NGS) is the most commonly used [55,59]. However, the Sanger sequencing method is still considered to be the gold standard [77]. DNA sequencing allows us to detect and identify both point mutations and small deletions/insertions [7].

Comprehensive analysis of MMR genes can be problematic due to their large size. Therefore, high throughput and massive parallel methods are required, such as next-generation sequencing (NGS) technology [85].

With the NGS method, it is possible to sequence the whole genome (WGS, whole-genome sequencing), whole exome (WES, whole-exome sequencing), or to perform targeted gene sequencing. The main advantage of NGS is the ability to detect single nucleotide variations (SNVs) or small insertions/deletions in several genes simultaneously [7,86,87]. Useful features include short time of analysis, detection of meager input of nucleic acids [86], and sensitivity and specificity reaching 99.9% for both parameters [77].

**Figure 4:**
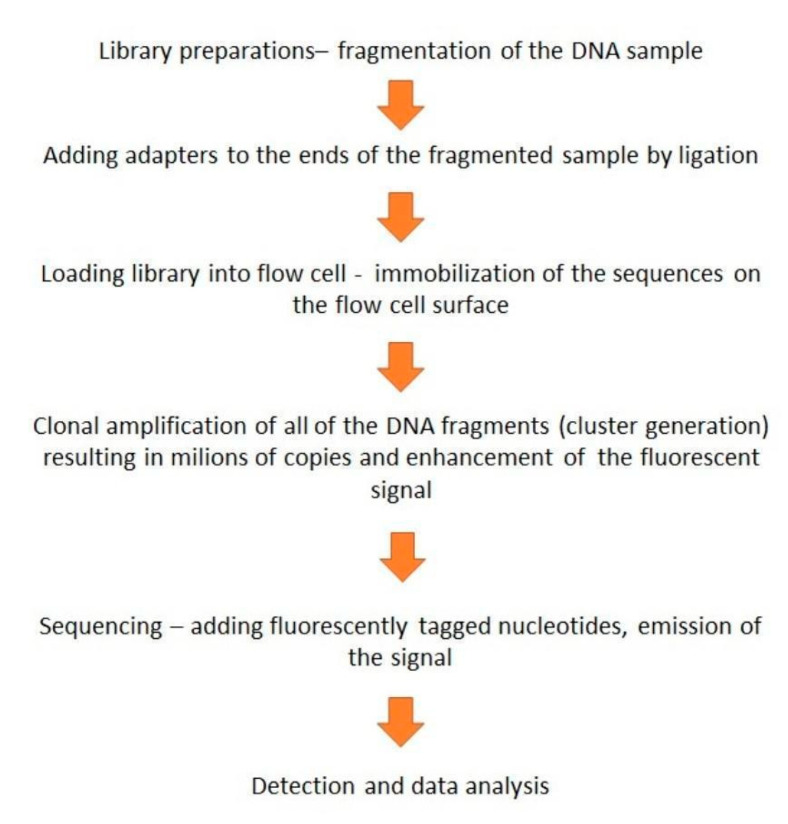
Schematically presented stages of next-generation sequencing (NGS) sequencing by synthesis based on Illumina’s method [88,89].

Targeted gene panels save time, reduce costs, and enable the exclusive analysis of areas of interest. Also, the sequencing is performed at a high depth, up to 500–1000× while in WGS the level of coverage is at 30–50×. The most commonly used type of NGS is sequencing by synthesis, the method developed by Illumina [88]. The process of sequencing is presented in Figure 4.

In LS diagnostics, commercial targeted gene panels, including exons and exon-intron regions of MMR/*EPCAM* genes, are available (e.g., HNPCC MASTR Plus, Agilent) [86]. However, it is also possible to design panels depending on needs. To simultaneously sequence several genes, a multiplex PCR is performed. Multiple libraries are pooled together and sequenced in the same run by adding discriminatory barcodes to each library [88]. Sanger sequencing as the gold standard is often performed to verify NGS results [77].

When no point mutation is detected, it is recommended to use methods that allow for the identification of more extensive rearrangements, deletions, and insertions, such as Southern blot, Array Comparative Genomic Hybridization (aCGH microarrays) or Multiplex Ligation-dependent Probe Amplification (MLPA) [7,55,62].

MLPA is the most commonly used method in LS diagnostics and makes possible the detection of significant structural mutations, such as genomic deletions, duplications or rearrangements of one or more exons [7], which represent from 5% to 20% of all of the MMR genes mutations [50]. In MLPA, instead of DNA sequencing, the probes are amplified. In the end, the peak pattern analysis indicates which of the sequences shows incorrect copy numbers. The method consists of 5–6 steps and was described in detail for the first time by Schouten et al. [90].

The results are presented as a ratio of 1.0 when both copies of the gene are noted in an examined sample, which means that each probe detected the same amount of the gene copies in both the tested and reference samples (no detected aberrations). A ratio of 0.5 indicates heterozygous deletion and 1.5 indicates heterozygous duplication. For LS, there are already designed sets of probes, which include *MLH1* and *MSH2* or all MMR and *EPCAM* genes [91,92]. It is also possible to study the level of methylation of genes using MS-MLPA (Methylation-Specific MLPA). This variant of the MLPA method can be used both for methylation profiling and copy number quantification. The performance of MS-MLPA is similar to a standard MLPA, except for the fact that the MS-MLPA generates two samples-one for copy number detection and one for methylation profiling, and the last one undergoes digestion with the HhaI enzyme, directly after ligation of the probes. Hybrids of probes and unmethylated DNA sequences are digested and do not generate a signal during capillary electrophoresis, unlike hybrids of probes and methylated samples [92,93]. With MS-MLPA, it is possible to detect hypermethylation of *MLH1* and *MSH2* genes. A summary of the advantages and limitations of molecular applications is presented in Table 6.

## 4. The Care and Treatment of Patients with LS and Their Families

The risk of developing cancer in individuals with LS before the age of 70 is approximately 80%. Therefore, it is crucial to identify patients with MMR/*EPCAM* mutations as soon as possible. This allows for the commencement of observation and care programs for affected individuals and their families. Early cancer detection will result in increased treatment efficiency and patients’ survival rates [13,52,102]. It is recommended for LS patients to attend genetic counseling before and after genetic testing to clarify any clinical, ethical, financial, or social issues that may arise during the diagnostics process. Also, educating the patient about the disease, the cancer risk, discussing the test results, and presenting further diagnostics or treatment options are also crucial factors. Patients diagnosed with LS should also be undergoing some form of psychological care [13,102].

Individuals with LS or high risk patients are usually recommended to perform a colonoscopy every 1–2 years starting at 20–25 years old if they carry mutations in *MSH2* or *MLH1* genes [13,52,66]. However, some studies indicate that colonoscopy surveillance once every two years might be more cost-effective than the annual approach [103,104]. In the case of an individual carrying a mutation in *MSH6* or *PMS2*, some sources advise considering the later inception of colonoscopy surveillance due to lower rates of CRC in these individuals [13,52,66,105]. In short, there are no formal or compelling guidelines for prophylactic colorectal surgery in LS [106]. 

Patients at high risk of LS should also be screened for extracolonic cancers. Several approaches are proposed for EC and ovarian cancer screening, such as pelvic examinations and endometrial sampling, starting at the age of 30–35 years every year, a transvaginal ultrasound or determination of CA-125 concentration in serum. Nevertheless, there is still a need for more evidence pointing to these screening methods’ influence on the mortality or cancer rate [13,52]. Prophylactic hysterectomy and salpingo-oophorectomy should also be considered for women with LS who have given birth or are in their 40s [13,52,56]. For gastric cancer screening, it is recommended to undertake esophagogastroduodenoscopy (EGD) with gastric biopsy at age 30–35 years and *H. pylori* testing at least [13,52,107]. Currently, the surveillance guidelines for the small intestine, urinary tract, pancreas, or prostate cancer in LS patients are still lacking or are limited [13,52].

Numerous studies suggest the role of aspirin in preventing cancer, but the evidence is not strong enough to make a recommendation for its regular use. Despite that, it might be suggested to consume 600 mg of aspirin daily for a minimum of two years to reduce LS-associated cancer risk [13,52]. It was also noted that dMMR tumors might respond differently to treatment, for example, 5-fluorouracil treatment. This type of chemotherapy is less effective, whereas irinotecan treatment is characterized by an increased response [5]. Furthermore, MMR-deficient ECs seem to be great candidates for immune checkpoint inhibitor therapies due to significantly increased PD-L1 (programmed cell death ligand) expression both in tumor and immune stromal, compared to carcinomas with sporadic *MLH1* hypermethylation [52,108,109]. Hence treatment with anti-PD-1 antibodies, such as pembrolizumab, has great potential, mainly in the treatment of ECs. Pembrolizumab was approved by the Food and Drug Administration (FDA) for the treatment of patients with unresectable/metastatic, MSI-H, or dMMR tumors [108,110,111]. EPCAM was also mentioned as it is correlated with worse survival, and metastasis is the target of therapy. The trifunctional anti-EpCAM with anti-CD3 antibody catumaxomab, under the trade name Removab, is used for intraperitoneal treatment for EPCAM-positive malignant ascites [112]. It should be noted that, additionally, other anti-EPCAM antibodies were investigated with good prognosis for their clinical implication in many cancers [113].

With different responses of MSI-H or dMMR tumors to several treatment approaches, optimizing a personalized treatment strategy for patients with LS-associated cancer might prove to be more promising and may result in decreased mortality. This highlights the need for the detection of individuals with LS.

## 5. Conclusions and Future Perspectives

The identification of individuals with Lynch syndrome has evolved in the past and continues to rapidly improve. The development of molecular testing allowed for the replacement of time-consuming and demanding screening methods, such as SSCP, DGGE, or DHPLC, with NGS technology. NGS makes it possible to sequence whole gene panels for many patients simultaneously, making the diagnostics process quick, accurate, and reliable. Also, MSI assay can be performed using the next-generation sequencing method and can easily be included in panels already used in molecular diagnostics, which would reduce the costs even further. Although NGS still has its limitations, there is no doubt that this method has revolutionized the whole diagnostics system. 

On the other hand, there is still a need for more accurate clinical criteria that would include all LS families. There are still unidentified LS families that were missed in the current approaches. Diagnostics should also be more focused on the identification of unaffected LS individuals rather than individuals with a newly developed tumor. This would lead to early detections and more frequent observations of people with MMR mutation and the introduction of a screening program, which in case of cancer development, will result in quick and more efficient treatment and thus increase the patient survival rate.

## Figures and Tables

**Table 1:** Characteristics of *MSH2*, *MLH1*, *MSH6*, and *EPCAM*.

Gene	Localization on Chromosome	Protein	Type of Mutations Leading to LS	Ref.
*MSH2* (human mutS homolog 2), 16 exons	2p21–p16.3	DNA binding domain, 2 domains interacting with MSH6/MSH3 and MutL homolog	large deletions (whole exons) approx. 30%, secondary epimutation due to the loss of 3′ end of the *EPCAM* gene	[32,41,42,43,44]
*MLH1* (human mutL homolog 1), 19 exons	3p22.2	3 domains (ATPase, MutS homologs, PMS2/MLH3/PMS1 interaction domain)	mainly missense, nonsense mutations, splicing aberrations and large rearrangements or constitutional epimutation resulting in hypermethylation of *MLH1* promoter	[32,34,45,46]
*MSH6* (human mutS homolog 6), 10 exons	2p16.3	an ATPase domain, a conservative sequence and adenine-repeats consisting motif	mostly missense or nonsense	[29,47]
*EPCAM* (epithelial cell adhesion molecule), 9 exons	2p21.2	intracellular domain (EpICD) regulating the expression of other genes responsible for growth, proliferation, migration, and differentiation of cell	deletion of the 3′ end resulting in the loss of termination sequence and production of *EPCAM-MSH2* hybrid transcript, and eventually resulting in hypermethylation of *MSH2* promoter	[29,41,44,48,49]

**Table 2:** The Amsterdam criteria I, II and the Revised Bethesda Guidelines [7,13,54].

**Amsterdam Criteria I:**
At least three relatives with histologically verified colorectal cancer and one of which is a first-degree relative of the other two *, At least two successive generations affected, At least one of the relatives with colorectal cancer diagnosed at < 50 years of age.
**Amsterdam Criteria II:**
At least three relatives with histologically verified HNPCC-associated cancer (colorectal cancer, endometrial, stomach, ovary, ureter/renal pelvis, brain, small bowel, hepatobiliary tract and skin (sebaceous tumors) and one of which is a first-degree relative of the other two *, At least two successive generations affected, At least one of the HNPCC-associated cancers should be diagnosed at < 50 years of age.
**Revised Bethesda Guidelines:**
CRC diagnosed at < 50 years of age, Presence of synchronous or metachronous CRC or other LS-associated tumors ** regardless of age, Colorectal cancer with MSI-H histology diagnosed in a patient < 60 years of age, Colorectal cancer or LS-associated * tumor diagnosed under the age of 50 years in at least one first-degree relative, Colorectal cancer or LS-associated tumor ** diagnosed at any age in two first- or second-degree relatives

* Familial adenomatous polyposis should be excluded. ** LS-associated tumors include a tumor of the colorectum, endometrium, ovary, pancreas, stomach, renal pelvis, ureter, brain, biliary tract, small bowel, sebaceous glands, and keratoacanthomas.

**Table 3:** Prediction models used to determine an individual’s risk for LS [13,59,60].

Prediction Model	Analyzed Criteria	Models’ Function	Sensitivity [%]	Specificity [%]
*MMRpredict*	Sex, age of CRC diagnosis, tumor location, synchronous or metachronous CRCs, EC in first-degree relatives and age of diagnosis	Calculating risk of carrying characteristics for Lynch syndrome mutations	69	90
*MMRpro*	Personal and family history of CRC and EC, age of diagnosis, if available—results of molecular testing for MMR genes	Calculating the risk of carrying germline mutations in any of the *MLH1/MSH2/MSH6* genes and risk of developing LS-associated cancer	89	85
*PREMM 1, 2, 6*	Sex, personal and family history of LS-associated cancers	Calculating the risk of carrying mutations in *MLH1/MSH2/MSH6* in the individual with suspected LS	90	67
*PREMM5*	Sex, age at genetic testing, personal and family cancer history	Calculating the risk of carrying mutations in *MLH1/MSH2/MSH6/PMS2/EPCAM* in the individual with suspected LS	89.4	49

**Table 4:** Immunohistochemistry (IHC) results and possible causes [13,63].

IHC Results	Interpretation
Retained MMR proteins expression	(a) When MSI-H tumor—germline mutation in MMR/*EPCAM* genes but possibly maintained protein expression (b) when MSI−L/MSS-sporadic cancer
Heterogeneity of MMR protein expression	If heterogeneity is observed despite proper performance of IHC, it might be reasonable to consider further molecular testing.
Loss of MSH2 protein expression	Germline *MSH2* mutation
Loss of MSH6 protein expression	Germline *MSH6* mutation, rarely *MSH2*
Loss of MSH2 and MSH6 protein expression	Germline MSH2/*EPCAM* mutation, rarely *MSH6*
Loss of MLH1 and PMS2 protein expression	Sporadic cancer or germline *MLH1* mutation—recommendation: further *BRAF/MLH1* methylation testing
Loss of MLH1 protein expression	Germline *MLH1* mutation
Loss of PMS2 protein expression	Germline *PMS2* mutation, rarely *MLH1*

**Table 5:** Gene scanning methods for unknown mutations and their mechanisms.

Method	The Mechanism/Application	Ref.
Single-Strand Conformation Polymorphism (SSCP)	single-strand nucleotide sequence under the non-denaturing conditions forms a unique conformation based on its primary DNA sequence, altered strand takes on a different conformation than a non-altered strand and shows different electrophoretic mobility, even a single nucleotide change can be detected, steps: amplification of targeted fragment, double-strand denaturation, non-denaturing polyacrylamide gel electrophoresis, and the analysis of the results	[80,81]
Denaturing Gradient Gel Electrophoresis (DGGE)	the different melting points of altered and unaltered double-stranded DNA at different concentrations of chemical denaturants steps: amplification of the targeted fragment through PCR, polyacrylamide gel electrophoresis with a rising concentration of the denaturing agent, and migration of double-stranded PCR products until a concentration of denaturing agent is equal to their melting point (Tm)	[80,81]
Denaturing High-Pressure Liquid Chromatography (DHPLC)	one of the chromatography methods for detection of single nucleotide substitution and small insertions/deletions separation of hetero- and homoduplexes on a chromatographic column under partial denaturation temperature conditions, using buffer gradients with TEAA and acetonitrile steps: DNA isolation and amplification of targeted sequence (approximately 150–600 bp) via PCR reaction; formation of hereto- and homoduplexes through DNA strand denaturation and reannealing caused by slow temperature reduction; elution (heteroduplexes are less stable and show less affinity for the column, and as a consequence are eluted earlier); monitoring of separation by measuring absorbance at 260 nm wavelength; detection and results presented as the chromatograms (homozygous sample—1 peak, the heterozygous—up to 4 peaks)	[82,83]
Conformation-sensitive Gel Electrophoresis (CSGE)	heteroduplex analysis for screening of mismatches in large multi-exon genes based on different electrophoresis mobility of hetero- and homoduplexes on a modified polyacrylamide gel	[84]
High-Resolution Melting (HRM)	detection of point mutations, small deletions/insertions, and large genomic rearrangements (qPCR-HRM) gene scanning technique performed on double-stranded DNA samples; the separation of the double-strand is monitored in real-time by a progressive change in fluorescence (due to the release of fluorescence dye from double-strand DNA denatured under temperature) the differences in the melting points distinguish altered and normal (wild type) alleles	[85]

**Table 6:** Comparison of selected diagnostics methods.

Method	Advantages, Applications	Disadvantages, Limitations
Next-generation sequencing (NGS)	sensitivity and specificity >99% [77], massively parallel sequencing in several genes simultaneously, a relatively short time of analysis, detection of low input of DNA samples [86,88], detection of SNVs and small insertions/deletions [94,95]	advanced bioinformatics systems and large data storage potential [96,97], filtering and data interpretation (various variants can be found when a large number or whole genes are sequenced) [94,97], issues with detecting structural rearrangements or copy number variations (CNVs) [95]
Multiplex Ligation-dependent Probe Amplification (MLPA)	wide diagnostic applications—copy numbers, point mutations detection, methylation profiling, also detected simultaneously, washing unbounded probes are not necessary, a simple and cost-effective method, easy analysis of the results [91,92]	does not detect balanced mutations, like balanced translocations or inversions (detects only ones which affect the probe binding sequence), probes can be designed only for known mutations—impossible to detect an unknown mutation, the heterozygous deletions analysis is reliable when tumor cells constitute 20–30% of the sample, heterozygous duplication—about 40% [91,92], does not provide precise deletion/insertion characteristics‘ [98]
High-Resolution Melting (HRM)	simple after proper optimization, fast, high-throughput, software supporting optimization available, relatively simple and not-expensive equipment needed [98,99]	detected variants not characterized, further characterization with another method, e.g., sequencing needed [98,99]
Sanger sequencing	the gold standard, mainly for detecting point mutations, high quality reads [98]	not cost-effective when a large number of samples and long sequences are analyzed, technically demanding method [98]
Single-Strand Conformation Polymorphism (SSCP)	detection of point mutations, deletions, and insertions, detection of unknown variants, simple and quite fast method [81]	low sensitivity and repeatability, amplicons not longer than 200–300 bp, detected variants not characterized, further characterization with another method, e.g., sequencing needed [80]
Conformation-sensitive Gel Electrophoresis (CSGE)	detection of single-nucleotide mutations, small insertions, and deletions, relatively high sensitivity and specificity, cost-effective [84]	detected aberrations need to be sequenced time-consuming method [84]
Denaturing Gradient Gel Electrophoresis (DGGE)	detection of unknown variants [80], relatively cheap, reliable heteroduplexes detection [98]	technically demanding, results must be characterized by another method, e.g., sequencing [80,98], GC-rich regions can be difficult to optimize and analyze [98,100]
Denaturing High-Pressure Liquid Chromatography (DHPLC)	sensitivity nearly 100% [82], a wide spectrum of applications: mutations and SNP detection, gene mapping, gene expression and methylation analysis [82,101], does not require modified primers or specific reagents [101], relatively cheap [81]	does not detect copy number aberrations [92], detected variants need to be characterized by sequencing, when more than one melting domain in tested amplicon-analysis of several temperatures required [82], chemical waste generation, not a high-throughput method [85]
Southern blot	detection of large insertions/deletions [82]	not always small deletions are detected [82] time-consuming [98]
